# Adhesion of Hydroxyapatite Nanoparticles to Dental Materials under Oral Conditions

**DOI:** 10.1155/2020/6065739

**Published:** 2020-05-05

**Authors:** Cíntia Mirela Guimarães Nobre, Norbert Pütz, Matthias Hannig

**Affiliations:** Clinic of Operative Dentistry, Periodontology and Preventive Dentistry, Saarland University Hospital, D-66421 Homburg, Saarland, Germany

## Abstract

Hydroxyapatite nanoparticles (nano-HAP) are receiving considerable attention for dental applications, and their adhesion to enamel is well established. However, there are no reports concerning the effects of HAP on other dental materials, and most of the studies in this field are based on *in vitro* designs, neglecting the salivary pellicle-apatite interactions. Thus, this *in situ* pilot study aims to evaluate the effects of three hydroxyapatite-based solutions and their interactions with different dental material surfaces under oral conditions. Hence, two volunteers carried intraoral splints with mounted samples from enamel and from three dental materials: titanium, ceramics, and polymethyl-methacrylate (PMMA). Three HAP watery solutions (5%) were prepared with different shapes and sizes of nano-HAP (HAP I, HAP II, HAP III). After 3 min of pellicle formation, 10 ml rinse was performed during 30 sec. Rinsing with water served as control. Samples were accessed immediately after rinsing, 30 min and 2 h after rinsing. Scanning electron microscopy (SEM) and transmission electron microscopy (TEM) were used to characterize the particles, and SEM evaluated the pellicle-HAP interactions. SEM and TEM results showed a high variation in the size range of the particles applied. A heterogeneous HAP layer was present after 2 h on enamel, titanium, ceramics, and PMMA surfaces under oral conditions. Bridge-like structures were visible between the nano-HAP and the pellicle formed on enamel, titanium, and PMMA surfaces. In conclusion, nano-HAP can adhere not only to enamel but also to artificial dental surfaces under oral conditions. The experiment showed that the acquired pellicle act as a bridge between the nano-HAP and the materials' surface.

## 1. Introduction

The application of hydroxyapatite nanoparticles (nano-HAP) in dentistry has received considerable attention in the past few years [1–4]. Hydroxyapatite (Ca_10_(PO_4_)_6_(OH)_2_) is a calcium-phosphate ceramic and the main mineral component of the dental enamel, the hard tissue on the exterior layer of a human tooth. This crystallite has a needle-like morphology and represents more than 90% of enamel mineral structure [5–7]. Synthetic nano-HAP are considered morphologically and structurally similar to the apatite crystals of enamel, revealing a high biocompatibility [1, 5, 6]. A recent literature review from Epple M. concluded that, when applied in adequate doses, HAP particles present no side effects to the human health, being a nontoxic and nonimmunogenic material [8]. Other characteristics that make it a desirable biomimetic material include high surface energy, high solubility, and optimal bioactivity [6, 8, 9].

Accordingly, nano-HAP has been increasingly employed for different dental applications. For instance, in restorative and preventive dentistry, HAP may be used to remineralize initial caries lesions on enamel, protecting teeth against caries and dental erosion [7, 10–13]. Given its properties, hydroxyapatite was added in various toothpaste and mouthrinse as an additional compound, not only to serve as a reparative material for damaged enamel but also as a polishing, whitening, and desensitizing agent [5, 7, 14–16]. Additionally, evidence on literature demonstrates that the size and shape of hydroxyapatite particles plays an important role, affecting the HAP properties and applications [17]. There are several kinds of raw synthetic HAP commercially available but, according to recent publications, those composed by smaller particles achieve better remineralizing effects [15, 16, 18, 19].

Although most of the literature confirmed these promising properties of hydroxyapatite nanoparticles, there are very divergent results [7]. While an increasing number of experiments revealed the potential of nano-HAP to repair enamel [1, 2, 19–21], other studies present no difference between the nano-HAP treatment and the standard fluoride treatment regarding the remineralization effects, some even showing less effective results [3, 11, 22]. These varying conclusions might be related to the methodology applied. Most studies regarding hydroxyapatite nanoparticles as an oral care product comprise *in vitro* designs, which give limited results. This method does not reproduce the real intraoral conditions, due to various individual-related factors, such as salivary flow, nutrition, or bacteria existent in the oral cavity [23].

Furthermore, most of the *in vitro* results show the direct interaction between HAP and the enamel surface [1, 2, 4, 17]. However, under oral conditions, a proteinaceous layer called acquired pellicle is immediately formed on any surface after exposure to the intraoral environment. The acquired pellicle is defined as an acellular and bacteria-free film composed of many salivary molecules, such as proteins, glycoproteins, mucins, immunoglobulins, lipids, bacterial components, and other macromolecules [23–25]. The pellicle acts as a protective barrier, has lubricant function, and also changes the free energy and charge of the material surface [25]. Thus, the pellicle-apatite interaction is the first step to understand the mechanisms behind the reported effects of the nano-HAP under oral conditions. Hence, an *in situ* design is the most appropriate method to evaluate such interaction, since it reproduces real-life oral condition, providing a more reliable result.

There are very few *in situ* studies concerning hydroxyapatite particles as an oral product, although they consistently show that HAP increases the protective effects of the pellicle against erosion [26], and that it can be used as remineralizing agent [27–29]. Recent *in situ* publications also showed that nano-HAP containing rinsing solutions were capable of reducing the initial bacterial adhesion on enamel without killing the bacteria. This is caused by an antiadherent effect against biofilm formation on enamel surfaces, thus having the potential to act as a biomimetic biofilm managing agent [9, 30]. However, additional *in situ*/*in vivo* studies are needed to clarify the HAP-pellicle interactions and the hydroxyapatite's mechanism of action.

As the pellicle is not restricted to enamel, another valuable question is whether the HAP particles would adhere to other dental materials, extending their benefits to artificial dental surfaces commonly used for oral rehabilitation. Thus, the aim of this *in situ* pilot study is to investigate if it is possible to adhere nano-HAP onto different dental materials' surfaces under oral conditions and to evaluate the effects of three different HAP powders. Additionally, we also assessed if there is any difference in the pellicle-HAP interactions between enamel and other dental substrates, such as titanium, polymethyl methacrylate resin, and ceramics.

## 2. Materials and Methods

This *in situ* pilot experiment was conducted in two healthy volunteers, aged between 30 and 35 years old. The inclusion criteria were good oral health with no signs of gingivitis, caries, or unphysiologically salivary flow rate [31]; no systemic diseases; no use of antibiotics or any kind of periodontal treatment within the past 6 months; nonsmoker; not pregnant or breastfeeding and absence of orthodontic appliances. The study protocol was approved by the Medical Ethics Committee of the Medical Association of Saarland, Germany (no. 283/03–2016), and an informed written consent was obtained from the subjects.

### 2.1. Tested Solutions

Hydroxyapatite nanoparticles (nano-HAP) in different sizes were used for the test solution (Table 1). The particle size was verified by scanning electron microscopy (SEM) and by transmission electron microscope (TEM). The containing HAP test solution was prepared mixing 0.5 g in 10 ml bidistilled water. Water rinse (10 ml) served as negative control. The subjects used the different rinsing solutions in different weeks to avoid interferences between tests and control solutions, preventing a possible crossover effect. According to our established protocol, the first solution used by each volunteer was the water control. One week later, the HAP test solutions were introduced respecting the following order: HAP I, HAP II, and HAP III; and respecting also a two weeks clearance period between each of them.

### 2.2. Samples

In addition to dental enamel, other common materials used for oral rehabilitation appliances were addressed: titanium (most used material on dental implants), feldspathic ceramics (used as dental restorative material), and polymethyl methacrylate resin (common material for the base of the prosthesis).

#### 2.2.1. Enamel Samples

Square enamel slabs measuring approximately 5 mm long and 1 mm thick were prepared from bovine incisors teeth. Later, they followed a standardized grinding procedure with grid sandpapers (from 240 to 2500 grit for SEM and TEM), and the smear layer was removed according to the following standardized cleaning procedure [30]. First, samples were washed with 3% NaOCl for 3 min, followed by ultrasonication with distilled water for 5 minutes. Afterwards, a disinfection in 70% ethanol for 15 minutes took place. And, finally, samples were washed with sterile water and stored at 4°C in sterile water for 24 h.

#### 2.2.2. Titanium Samples

Titanium (Ti) discs microstructured surface by sandblasting and acid etching (SLA) with Ra = 2 *μ*m, grade 2, were obtained from Dentsply Implant Systems Sirona, Mannheim, Germany (diameter 5 mm, height 1 mm). Ti discs were polished by wet grinding with abrasive paper (800 to 4000 grit). To remove the resulting smear layer and for disinfection purposes, Ti discs were immersed in isopropanol (70%) for 10 min, followed by washing in distilled water.

#### 2.2.3. Ceramic Samples

Square/rectangular ceramic slabs measuring approximately 5 mm long and 1 mm thick were cut from feldspathic ceramic blocks (VITABLOCS Mark II from VITA Zahnfabrik, Germany). These slabs were polished with grit sandpapers (from 240 to 4000 grit). For cleaning and disinfection purposes, the ceramics samples were immersed in isopropanol (70%) for 15 min, followed by a wash in distilled water.

#### 2.2.4. PMMA Samples

Polymethyl methacrylate (PMMA) resin with 5 mm diameter and 1 mm thick was prepared by a prosthetic technique from Universität des Saarlandes with an autopolymerizing prosthetic resin kit (powder and monomer) from Paladent® (Kulzer, Germany) in accordance with the manufacturer's instructions. Polishing was performed with grid sandpapers (1200 to 4000 grit), and for cleaning purposes, the following protocol was applied. The samples were placed three times (10 min each) in the ultrasonicator, two times with isopropanol 70%, and one with sterile water. Finally, the samples were dried before attaching to the splints.

### 2.3. Oral Exposure

**Figure 1:**
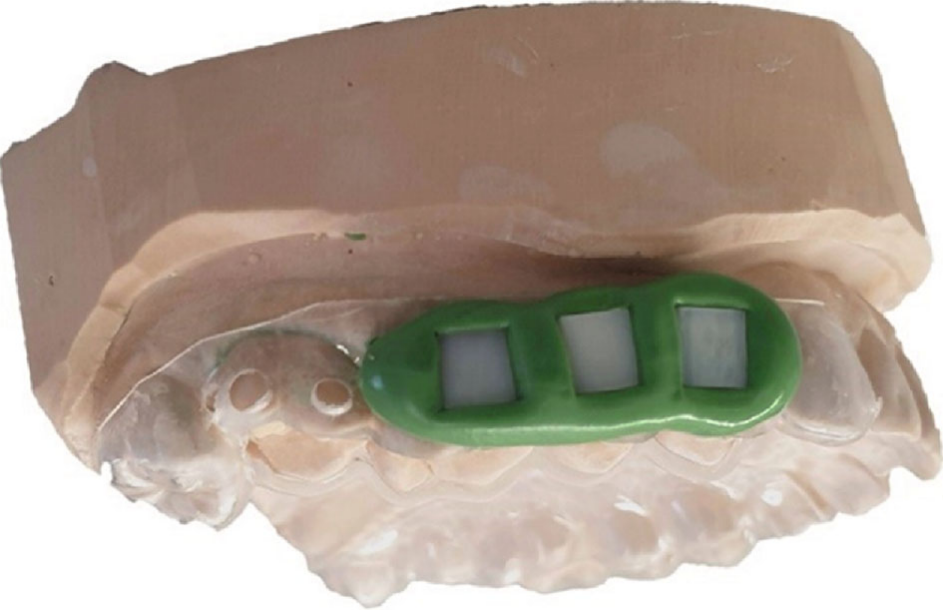
Maxillary intraoral splints with mounted enamel slabs.

To evaluate the possible interaction between the hydroxyapatite particles and the acquired pellicle, the samples were mounted in customized maxillary splints (Figure 1). They were prepared from 1.5 mm thick methacrylate foils, extending from premolars to the first molar. Perforations in the buccal aspects of the splints were prepared to fix the polyvinyl siloxane impression material, in which each material was placed.

Before the use of the splints, the volunteers brushed the teeth without toothpaste and rinsed with tap water only to avoid possible interferences from the compounds of the toothpaste. After the splints were placed intraorally with 3 samples on each side (total of 6 samples of the selected material), a one-time rinse (30 s) of 10 ml of the selected solution (HAP I, HAP II, HAP III, or water) was performed after a 3 min of pellicle formation. One sample from each side was removed immediately after rinse, 30 min and 120 min after rinse. After each removal, the samples were rinsed with distilled running water to remove nonadsorbed particles. Then, samples were prepared for scanning electron microscopy (SEM). Each volunteer used the splint for two hours, from 10 h to 12 h. The use of the splint carrying 6 samples was repeated for each of the 4 materials and each of the 4 tested solutions, giving a total of 96 samples per volunteer.

### 2.4. Scanning Electron Microscopy

In order to characterize particle size and shape of the different powders, HAP I, HAP II, and HAP III solutions were directly applied to SEM sample holder (aluminium plate) and analyzed by SEM and energy-dispersive X-ray spectroscopy (EDX) evaluations in a XL30 ESEM FEG (FEI, Eindhoven, The Netherlands) at 5 kV and 10 kV at 20,000-fold magnification.

Intraorally exposed samples were prepared for SEM analysis to investigate the pellicle coverage at different times after rinsing and its relationship with hydroxyapatite particles. After oral exposure, samples were washed with sterile water followed by a fixation with 1 ml 2% glutaraldehyde in 0.1 M cacodylate buffer during 2 h at 4°C. Finally, the specimens were left to airdry overnight at room temperature in the air chamber. The next day, samples were sputter-coated with carbon and analyzed by SEM and energy-dispersive X-ray spectroscopy (EDX) evaluations in a XL30 ESEM FEG (FEI, Eindhoven, The Netherlands) at 5 kV and 10 kV, consecutively, at up to 20,000-fold magnification.

### 2.5. Transmission Electron Microscope

In order to evaluate the particle's sizes, 5% nano-HAP solutions were prepared from each powder and directly applied on Pioloform-coated copper grids and analyzed on TEM Tecnai 12 Biotwin (FEI, Eindhoven, The Netherlands) under a magnification up to 100.000-fold.

Additionally, a supplementary experiment was performed to validate the presence of the pellicle layer. The volunteers carried the same splints with only 2 enamel specimens (one each side) and performed a one-time 30s-rinse with 10 ml of HAP II solution after 3 min of pellicle formation. The samples were removed from the oral cavity 30 min after rinsing. Immediately after volunteers took off their splints, the samples were washed with sterile water to remove not adhered bacteria. The specimens were placed in 1,5 ml tubes with 1 ml 1% Glutaraldehyde fixing solution at 4°C during 1 h. After primary fixation, samples were washed with cacodylate buffer 0.1 M 4 times, 10 min each, and stored at 4°C in cacodylate buffer. Samples were fixed in osmium tetroxide during 1 h in a dark chamber at room temperature, followed by 5 times of 10 min wash in distilled water and immersion in 30% ethanol overnight. Following the TEM preparation procedures, dehydration was performed at room temperature. The samples passed through series of 50% (2x 10 min), 70% (2x 20 min), 90% (2x 30 min), and 100% (2x 30 min) ethanol. Finally, they were further immersed in 100% acetone 2 times, 30 min each, and stored overnight in an acetone/Araldite (Agarscientific, Stansted, United Kingdom) mixture plus 3% accelerator (mixture A) at room temperature. On the following day, mixture A was poured out and a second mixture, mixture B was prepared (Araldite mixture with 2% accelerator). Samples were left again overnight in mixture B at room temperature in the air chamber. Next, a new mixture B was used to fill the embedding forms. Then, samples were incubated for polymerization for 48 h at 65°C.

Finally, ultrathin sections were cut in an ultramicrotome with a diamond knife (Leica EM UC7, Germany) and mounted on Pioloform-coated copper grids and contrasted with aqueous solutions of uranyl acetate and lead citrate at room temperature. After an intensive wash with distilled water, biofilms could be analyzed with a TEM Tecnai 12 Biotwin (FEI, Eindhoven, The Netherlands) under a magnification up to 100.000-fold.

## 3. Results and Discussion

This pilot study provided, for the first time, evidence that it is possible to accumulate round- and crystallite-shaped nano-HAP on the natural enamel surface, as well as on different artificial dental surfaces under oral conditions, and the pellicle may play an important role on the particles' adhesion mechanism.

### 3.1. Enamel Samples

In this study, bovine enamel was applied. The use of human teeth for research has decreased over the last decades due to several factors, such as difficult standardization, difficulty to obtain sufficient amounts, and mostly due to ethical issues [32, 33]. Hence, the use of animal teeth came as an alternative. Bovine enamel has similarities with human enamel regarding mineral composition, density, and structure. Bovine teeth can be easily obtained on a large scale, and they have more uniform characteristics. Additionally, because of their bigger size and flatter surface, they are also easy to handle [4, 32, 34].

### 3.2. Hydroxyapatite Particles

**Figure 2:**
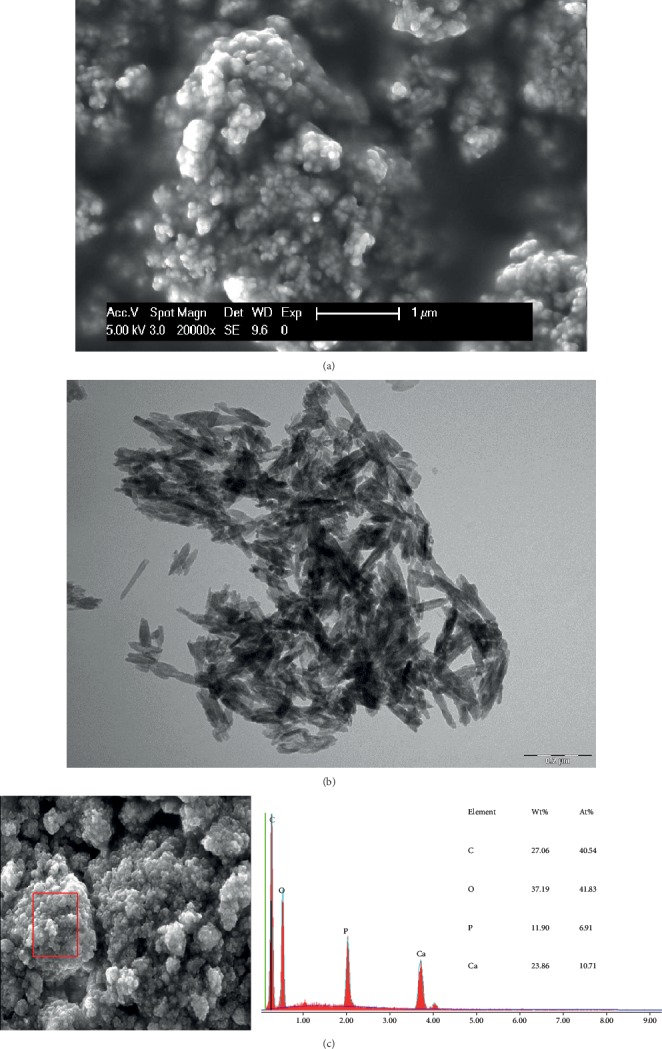
SEM figure at 20,000-fold (a), TEM figure at 68,000-fold (b), and EDX analysis at 5,000-fold (c) magnification from HAP I powder immersed in water solution. The images reveal the crystallite shape and the tendency for clusters formations.

**Figure 3:**
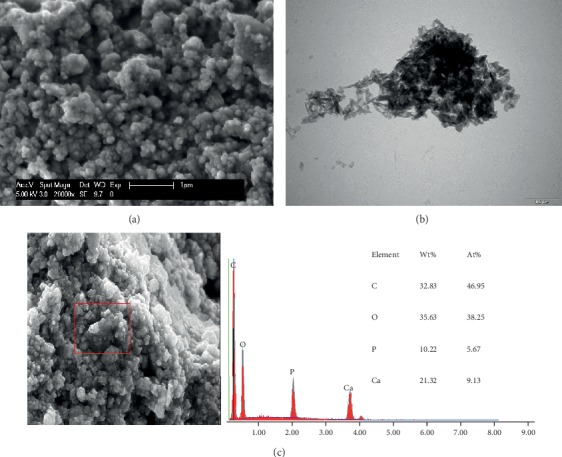
SEM figure at 20,000-fold (a), TEM figure at 68,000-fold (b), and EDX analysis at 5,000-fold (c) magnification from HAP II powder immersed in water solution. The images reveal the crystallite shape and the tendency for clusters formations.

**Figure 4:**
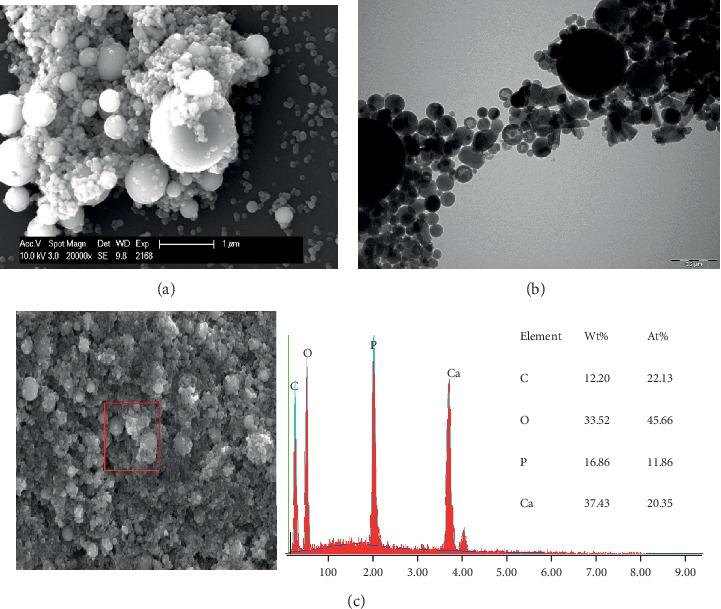
SEM figure at 20,000-fold (a), TEM figure at 68,000-fold (b), and EDX analysis at 5,000-fold (c) magnification from HAP III powder immersed in water solution. Individual particles can be easily detected.

Previously published studies demonstrate that the particles' and aggregates' size and shape affect the HAP properties and its applications, and the particle size is a key factor for adhesion on enamel [4, 15, 16, 18]. In an *in vitro* study, Li et al. proposed that crystallites of HAP with a 20 nm diameter would provide the best size for nano-HAP applications [17]. In the present study, three different hydroxyapatite powders, containing particles with a median size in the nanoscale were evaluated, hypothesizing that smaller nanoparticles (HAP I) would have better adhesion results. The particles' size of each hydroxyapatite powder was analyzed with SEM and TEM. HAP I and HAP II had a small size range, presenting a certain uniformity in the size of its particles. HAP I had particles ranging from 30 to 70 nm (Figure 2), while HAP II particles ranged from 60 to 120 nm (Figure 3). HAP III had the largest size variation, ranging from approximately 50 nm to particles even bigger than 1 *μ*m (Figure 4). These variations on the particles' sizes of each hydroxyapatite powder applied in this study revealed a certain similarity between them. Therefore, the size influence of apatite particles on their adhesion behavior under oral conditions could not be evaluated in detail in this study. The present findings indicate that better methods for HAP synthetization should be performed to standardize the particle' size.

Concerning the shape characteristics of HAP, the present SEM and TEM analysis confirmed the crystallite-like structures of the HAP I and HAP II particles (Figures 2 and 3). Figure 4 shows a majority of round-shaped particles for HAP III, but on the TEM picture, nonglobular structures are also present. Thus, HAP III is composed by rounded and a few needle-like particles. Interestingly, the round particles in this experiment adhered to each surface in a similar way as the needle-like particles. *In vitro* experiments revealed that HAP crystallites would have better adhesion to enamel because they have a morphology similar to natural enamel building units [4, 17]. But another *in vitro* study showed that spherical nano-HAP had a great potential to remineralize the enamel [1]. To our knowledge, this is the first *in situ* study comparing the effects between spherical and crystallite-like nano-HAP, and it showed that both can adhere to the pellicle formed *in situ* on different dental materials.

**Figure 5:**
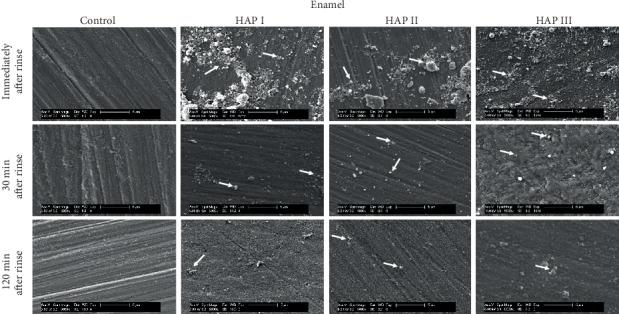
SEM micrographs at 5,000-fold magnification of the pellicle and the nano-HAP particles on enamel samples. The pellicle formation and the hydroxyapatite particles are visible at three different time-points: immediately, 30 min and 2 h after mouthwash with 5% HAP I, HAP II, and HAP III. White arrows point to HAP particles accumulated onto the enamel surface.

**Figure 6:**
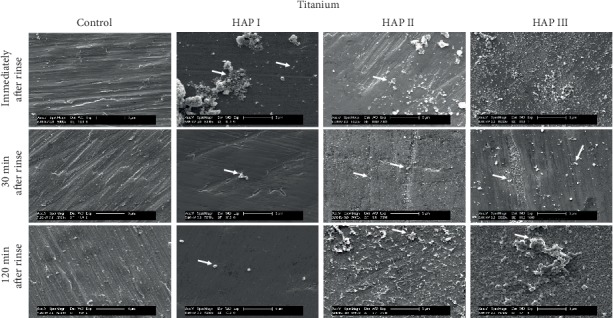
SEM micrographs at 5,000-fold magnification of the pellicle and the nano-HAP on titanium samples. The pellicle formation and the hydroxyapatite particles are visible at three different time-points: immediately, 30 min and 2 h after mouthwash with 5% HAP I, HAP II, and HAP III. White arrows point to HAP particles accumulated onto the titanium surface.

**Figure 7:**
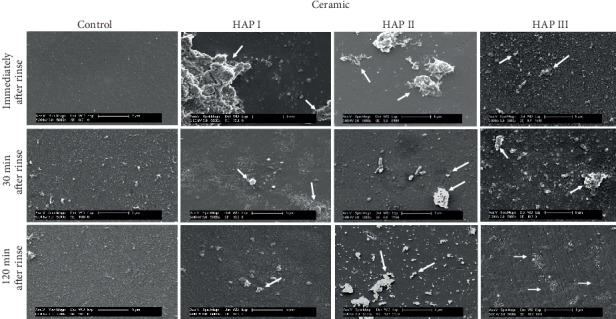
SEM micrographs at 5,000-fold magnification of the pellicle and the nano-HAP on ceramic samples. The pellicle formation and the hydroxyapatite particles are visible at three different time-points: immediately, 30 min and 2 h after mouthwash with 5% HAP I, HAP II, and HAP III. White arrows point to HAP particles accumulated onto the ceramic surface. HAP III tend to accumulate on surface irregularities after 2 h (white asterisks).

**Figure 8:**
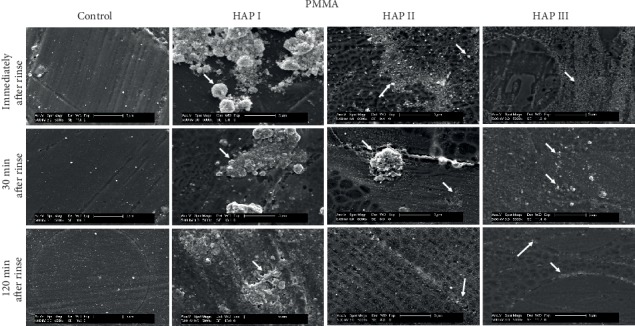
SEM micrographs at 5,000-fold magnification of the pellicle and the nano-HAP on PMMA samples. The pellicle formation and the hydroxyapatite particles are visible at three different time-points: immediately, 30 min and 2 h after mouthwash with 5% HAP I, HAP II, and HAP III. White arrows point to HAP particles accumulated onto the PMMA surface.

Additionally, regardless of their different sizes and shapes, all of the three tested hydroxyapatites had the tendency to agglomerate in aqueous solution (Figures 2–4), forming aggregates of different sizes and irregular shapes. Individual particles were more frequently seen in the HAP III solution, presenting a clear round-shaped conformation. HAP I and II rarely allowed observing individual crystallites when in watery solution. This propensity to aggregate might be related to the characteristic of hydroxyapatite being a dipole molecule and consequently to the Van der Waals and electrostatic forces in between the particles [30]. Another common result for all tested solutions was that the quantity of hydroxyapatite particles and the clusters' size on each material surface decreased over time (Figures 5–8). This pattern can be explained by the continuous process of adsorption and desorption that takes place in the oral cavity [25]. After rinsing with the HAP solutions, the particles and clusters were deposited on the material surface. Over time, those particles are dissolved in the saliva. Thereby individual intraoral shearing forces help to disrupt them into small aggregates and single particles, where some of them are readsorbed to the pellicle and others are swallowed. The continuous dissolution of hydroxyapatite nanoparticles also takes place and intensifies these results. Additionally, bigger clusters are more susceptible to shearing forces, removal from the material surface, and later swallowing [4, 16].

### 3.3. Hydroxyapatite Adhesion Efficacy

**Figure 9:**
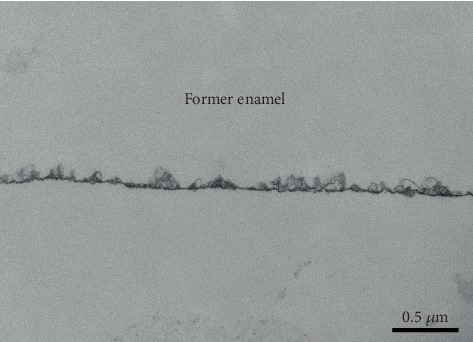
TEM micrograph at 30,000-fold magnification revealing the presence of the 30 min pellicle on enamel sample rinsed with HAP II solution. The pellicle manifests itself as continuous electron-dense layer. The enamel was dissolved due to demineralization of the specimens and thus is no more visible in the TEM figure.

SEM micrographs from the samples rinsed with water (control) (Figures 5–8) showed a thin and uneven film with globular structures on all surfaces after three minutes of intraoral exposure. In order to verify the existence of a pellicle layer on the specimens' surface TEM-analysis of selected samples was performed. The TEM figure (Figure 9) verifies the presence of the acquired pellicle on the specimens. After 30 min, the pellicle coverage increased on all four tested materials, leading to an almost fully knotted surface after 2 h, composed by salivary protein aggregates with globular shape. Thus, the acquired pellicle formation in the control experiment progressed in accordance with the literature, which states that the adsorption of proteins and consequent formation of salivary pellicle starts within seconds after a material is exposed to oral cavity environment [25]. Protein-protein interactions continue, and a thicker and more homogeneous proteinaceous film is visible after 30 and 120 minutes. Thus, the amount of absorbed proteins increased over time in all tested samples. Literature indicates that the acquired pellicle growth reaches a plateau after 30-90 min, reaching its full thickness around one or two hours [25].

When the 5% nano-HAP solutions were applied, the nanoparticles were randomly distributed on all samples' surfaces (Figures 5–8). Immediately after the mouthwash, most of the samples presented their surface heterogeneously covered with particles and clusters with large variations of size and shapes. HAP I and HAP II yielded large cluster structures with sizes up to 4 *μ*m. HAP III had a better coverage distribution on all materials surfaces immediately after rinse. Thirty minutes after rinsing, the number of adhered particles decreased, and more disperse particles could be seen on SEM; however, smaller clusters were also visible. Surprisingly, almost all nanoparticles were washed out on titanium samples rinsed with HAP I. Finally, 2 h after rinsing, even smaller particles were visible on SEM figures (Figures 5–8) and small clusters could still be found, but particles and clusters larger than 1 *μ*m were rarely seen. The amount and the size of the hydroxyapatite clusters on each material surface decreased over time. The influence of HAP particle sizes was also reported by Jin et al. in an *in vitro* experiment, showing that particles in a nanometer range (<1 *μ*m) had better adhesion to enamel [16]. The *in situ* study from Kensche et al. also revealed only isolated microclusters 2 h after mouthrinsing [30]. Thus, due to the larger contact area, smaller particles and clusters would better adhere to the pellicle components.

In summary, after 2 h of intraoral exposure, all samples presented a scattered and heterogeneous layer of nano-HAP in contact with the pellicle outer layer. A homogeneous coverage could not be achieved, probably due to the complex interaction that occurs in the intraoral environment, or due to the shape and size of the selected particles. Until now, the scarce *in situ* literature on this topic reported only the presence of adhesion between the HAP particles and the enamel surface [9, 30]. But based on the present results, it is possible to conclude that the adhesion of hydroxyapatite nanoparticles also occurs on other dental surfaces as well, such as titanium, ceramics, and polymethyl methacrylate resin, opening a promising research field in preventive dentistry. This study, however, presented some limitations. The stability of these adhesion interactions of the apatite particles on the different tested surfaces was not evaluated over longer periods of time. Another clear limitation was the number of volunteers, which made it not possible to perform a quantitative analysis. The reasons behind this choice are related to the increased number of times that each volunteer had to use the intraoral appliance. Therefore, more *in vivo*/*in situ* experiments with a higher number of subjects need to be performed to elucidate the molecular mechanisms behind the pellicle-apatite interactions.

The physical and chemical characteristics of each material influence the pellicle and biofilm formation and might also have an influence on HAP deposition [35]. Comparisons between all materials showed that ceramic samples had the smallest quantity of HAP accumulated after 2 h (Figure 7), while the PMMA samples presented higher numbers of particles and clusters after the same time (Figure 8). The porosity and retention areas from PMMA explains its association with higher HAP deposition [36, 37]. Concerning the HAP solution used, HAP II and III were more effective in covering titanium samples, and hydroxyapatite particles from HAP I were almost absent. Similar coverage is visible between the three solutions on enamel and on PMMA surfaces. Figure 7 shows that particles from HAP III were also accumulated on surface irregularities of ceramics caused by the polishing process. Thus, regardless of the round or crystallite shape and size variations, all three tested HAP nanoparticles could adhere to pellicle-covered enamel, titanium, ceramics, and PMMA.

**Figure 10:**
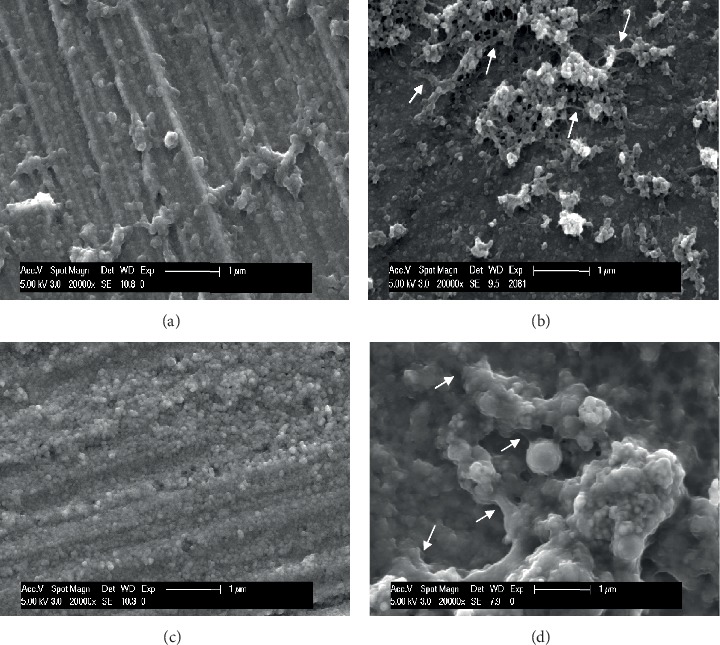
SEM micrographs at 20,000-fold magnification showing the 2-h pellicle on titanium (a) and enamel (c) control samples in comparison with titanium rinsed with HAP II (b) and enamel rinsed with HAP III (d). Higher magnification figures provide the visualization of connective structures between HAP II solution and titanium surface (b) and HAP III and enamel surface (d) 2 h after oral exposure to these HAP solutions. The HAP particles are in direct contact with globular structures from the acquired pellicle. Arrows point to connective bridges in-between the HAP particles and between them and the pellicle.

Higher SEM magnification was used to analyze the interactions between the accumulated nano-HAP and the pellicle that covered each substrate surface. Interestingly, when the hydroxyapatite containing solutions were applied, it was possible to observe connective structures between the particle and the pellicle formed on enamel, titanium, and PMMA surfaces after 2 h of oral exposure to the respective rinses (Figure 10). On ceramics, there was no possibility to find these connective structures due to reduced quantity of HAP on this surface after 2 h. According to Vukosavljevic et al., pellicle precursors proteins, such as histatins or statherins have a high affinity to hydroxyapatite crystals present on natural teeth, starting the acquired pellicle formation process [38]. Thus, the adhesion of the synthetic nano-HAP could be related to these pellicle components, which act as a bonding agent. More *in vivo*/*in situ* studies should be performed to clarify these relationships in detail.

## 4. Conclusions

It was presented for the first time that, regardless of the shape and size, all nano-HAP applied as oral rising solutions presented good, but heterogeneous coverage on enamel, titanium, ceramic, and PMMA surfaces under oral condition. Therefore, nano-HAP can adhere not only to enamel but also to artificial dental surfaces under oral conditions. The pilot experiment revealed that in the presence of the acquired pellicle, a bridge between the nano-HAP and the materials' surface could be observed.

Given the increasing importance of HAP nanoparticles in dentistry, it is significant to understand the mechanism of interaction of these particles with the intraoral environment. Despite the interesting results obtained in this pilot study, its limitations imply the need for more experiments with a higher number of subjects to better elucidate the pellicle-apatite interactions.

## Figures and Tables

**Table 1:** Specification of hydroxyapatite particle powders according to manufacturer's information.

	Company	Country	Median size	Configuration
HAP I	Eprui	China	40 nm	Needle
HAP II	Kalichem	Italy	100 nm	Needle
HAP III	Sigma Aldrich	Germany	< 200 nm	Spherical

## Data Availability

All data supporting the results are in the manuscript.
